# Dynamic changes in clonal cytogenetic architecture during progression of chronic lymphocytic leukemia in patients and patient-derived murine xenografts

**DOI:** 10.18632/oncotarget.17432

**Published:** 2017-04-26

**Authors:** Nicholas J. Davies, Marwan Kwok, Clive Gould, Ceri E. Oldreive, Jingwen Mao, Helen Parry, Edward Smith, Angelo Agathanggelou, Guy Pratt, Alexander Malcolm R. Taylor, Paul Moss, Mike Griffiths, Tatjana Stankovic

**Affiliations:** ^1^ Institute of Cancer and Genomic Sciences, University of Birmingham, Birmingham, UK; ^2^ West Midlands Regional Genetics Laboratory, Birmingham Women's NHS Foundation Trust, Birmingham, UK; ^3^ Institute of Immunology and Immunotherapy, University of Birmingham, Birmingham, UK

**Keywords:** chronic lymphocytic leukemia, xenograft, clonal evolution, multiplexed-FISH, cytogenetics

## Abstract

Subclonal heterogeneity and clonal selection influences disease progression in chronic lymphocytic leukemia (CLL). It is therefore important that therapeutic decisions are made based on an understanding of the CLL clonal architecture and its dynamics in individual patients. Identification of cytogenetic abnormalities by FISH remains the cornerstone of contemporary clinical practice and provides a simple means for prognostic stratification. Here, we demonstrate that multiplexed-FISH can enhance recognition of CLL subclonal repertoire and its dynamics during disease progression, both in patients and CLL patient-derived xenografts (PDX). We applied a combination of patient-specific FISH probes to 24 CLL cases before treatment and at relapse, and determined putative ancestral relationships between subpopulations with different cytogenetic features. We subsequently established 7 CLL PDX models in NOD/Shi-SCID/IL-2Rγc^tm1sug^/Jic (NOG) mice. Application of multiplexed-FISH to these models demonstrated that all of the identified cytogenetic subpopulations had leukemia propagating activity and that changes in their representation during disease progression could be spontaneous, accelerated by treatment or treatment-induced. We conclude that multiplexed-FISH in combination with PDX models have the potential to distinguish between spontaneous and treatment-induced clonal selection, and therefore provide a valuable tool for the pre-clinical evaluation of novel therapies.

## INTRODUCTION

Genomic aberrations play an important pathogenic role in chronic lymphocytic leukemia (CLL), by shaping its clinical course and response to treatment [1]. It was recognized as early as the 1990s that certain cytogenetic aberrations confer differential clinical outcomes, culminating in the hierarchical classification scheme devised by Döhner and colleagues that continues to be of major clinical relevance to this day [2]. In recent years, next generation sequencing (NGS) has allowed identification of recurrent genetic mutations, providing an additional dimension to the CLL genomic landscape [3–8]. However, despite the informative potential of NGS technology, identification of cytogenetic abnormalities by FISH remains the cornerstone of contemporary clinical practice, and continues to provide a validated, simple and cost-effective means of stratifying patients into distinctive prognostic subgroups who could benefit from different therapeutic approaches.

Among the most frequent cytogenetic lesions in CLL, del(17p) is associated with the worst prognosis, followed by del(11q). Trisomy 12 and del(6q) confer intermediate risk, while patients with normal karyotype or isolated del(13q) have the most favorable outcome [9–12]. Del(17p) and del(11q) are enriched in patients relapsing from chemotherapy or chemoimmunotherapy, which may reflect chemotherapy-driven selection of aggressive subclones containing these cytogenetic aberrations [12–17]. Equally, cytogenetic abnormalities are clinically significant in the context of targeted therapies such as the B-cell receptor (BCR) signaling inhibitor ibrutinib, with the majority of relapsing cases featuring del(17p), del(11q) and/or complex metaphase cytogenetics [18, 19].

Given the clinical importance of cytogenetics in CLL, it is relevant to ask how cytogenetic aberrations evolve, either spontaneously or under different treatments. Multiplexed-FISH, which involves the simultaneous use of multiple FISH probes corresponding to different chromosomal regions of interest, permits resolution of subclonal complexity and assessment of the cytogenetic composition at the single-cell level [20]. Longitudinal tracking of cytogenetic abnormalities by multiplexed-FISH may therefore provide potentially valuable information on their evolutionary pattern in individual patients under the influence of different treatments. This is not possible with comparative genome hybridization or single-nucleotide polymorphism array-based methodologies that are carried out on bulk cellular populations [21, 22].

Importantly, the application of multiplexed-FISH in CLL patient-derived xenograft (PDX) models has the potential to enhance their utility as tools for pre-clinical drug evaluation. Anderson and colleagues demonstrated the use of multiplexed-FISH to interrogate subclonality not only in samples from patients with acute lymphoblastic leukemia (ALL) but also in ALL PDX, allowing delineation of the composition of cytogenetic abnormalities in leukemia propagating cells [20]. Given the relatively limited number of recurrent cytogenetic abnormalities in CLL, we reasoned that multiplexed-FISH would be ideally suited to investigate whether distinct CLL subpopulations containing different combinations of cytogenetic aberrations can be recapitulated in PDX models as observed in acute leukemias. Moreover, we reasoned that modeling cytogenetic aberrations in PDXs would enable assessment of the impact of different treatments on the clonal cytogenetic architecture. In light of the multitude of novel therapeutic agents currently under investigation for CLL and the trend towards precision medicine, *in vivo* modeling of clonal selection and dynamics in PDXs could be invaluable in informing therapeutic stratification.

In this study, we demonstrate the functional utility of multiplexed-FISH in CLL. The combinations of cytogenetic aberrations in 24 untreated CLL samples were established at single-cell resolution by multiplexed-FISH from which putative ancestral relationships between CLL subpopulations with different cytogenetic features were established. In selected cases, we also analyzed sequential samples to assess the impact of treatment upon the composition of cytogenetic aberrations at the single-cell level. In addition, multiplexed-FISH analysis of PDX models was employed to interrogate the leukemia propagating activity of distinct CLL subpopulations carrying different combinations of cytogenetic lesions. Finally, and most importantly, we demonstrated that changes in the CLL cytogenetic architecture, both spontaneous and treatment-induced, can be modeled in PDXs.

## RESULTS

### Multiplexed-FISH provides a single-cell resolution snapshot of the CLL cytogenetic architecture

**Figure 1:**
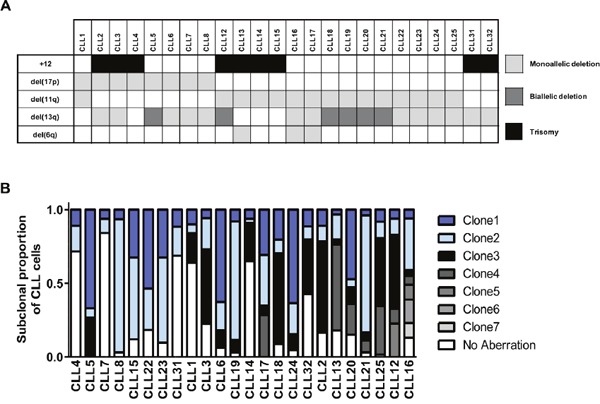
Multiplexed-FISH reveals cytogenetic subclonal heterogeneity in CLL **(A)** From 128 CLL patients, these 24 pre-treatment CLL samples were identified as amenable for multiplexed-FISH based on the presence of del(6q), del(11q), del(13q), del(17p) and trisomy 12 in various combinations. **(B)** Multiplexed-FISH with patient-specific probe combinations revealed the cytogenetic architecture of each of the 24 CLLs. The size of each clone is expressed as a proportion of the total number (≥200) of cells analyzed. The samples are arranged according to the cytogenetic complexity, with those having fewer different subclones placed on the left side of the figure. The most complex subclones are presented at the top of each bar. +12 indicates trisomy 12.

Initially, we screened untreated CLL samples from 128 individuals for the presence of clinically relevant cytogenetic lesions, namely del(11q22.3), del(17p13.1), del(13q14.3), del(6q23.3) and trisomy 12, and identified a cohort of 24 patients with at least two cytogenetic abnormalities (Figure 1A). This cohort was enriched for the presence of del(11q) and del(17p), two cytogenetic abnormalities that are frequently associated with a complex karyotype.

We then performed multiplexed-FISH using combinations of FISH probes specific for each patient and based upon the cytogenetic aberrations detected in the initial round of single probe FISH (Supplementary Table 2). Two hundred cells were assessed for the simultaneous presence of patient specific probes, and the different combinations recorded. The cohort analyzed revealed varied genetic heterogeneity in relation to these markers, with samples displaying 3 to 8 distinct cytogenetic combinations (Figure 1B). Thus, our results demonstrate the ability of multiplexed-FISH to detect multiple CLL subpopulations containing distinct combinations of clinically relevant cytogenetic lesions, providing a snapshot of the cytogenetic architecture at single-cell resolution.

### Multiplexed-FISH reveals distinct evolutionary patterns of cytogenetic aberrations under different treatments

**Figure 2:**
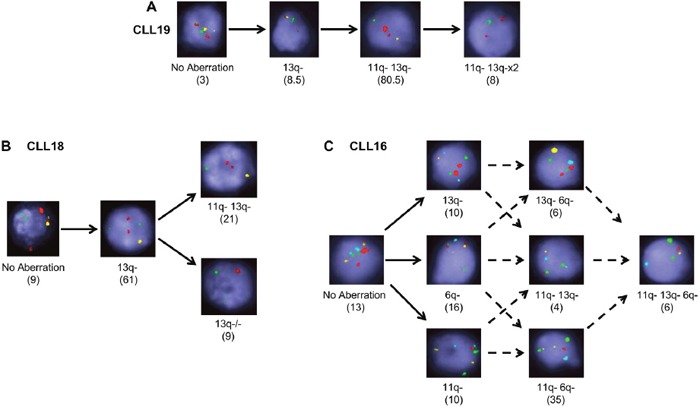
Multiplexed-FISH resolves both branching and linear evolution in CLL Multiplexed-FISH analysis enables establishment of the evolutionary history of a sample revealing that CLL populations undergo either **(A)** linear, **(B)** simple branching or **(C)** complex branching patterns of evolution. Original magnification ×100. Solid arrows denote the likely trajectory of evolution; dashed arrows denote possible trajectories. Numbers in brackets denote percentage of cells in that subclone. 6q- indicates del(6q); 11q-: del(11q); 13q-: del(13q); 13q−/−: del(13q)x2. Probe combinations were **(A, B)** 13q: yellow; 11q: green; 17p: red; **(C)** 13q: yellow; 6q: aqua; 11q: red; CEP11 (chromosome 11 control): green.

As multiplexed-FISH enables identification of multiple cytogenetic lesions in single cells, we used this information to infer the temporal order of the acquisition of cytogenetic lesions and to decipher the likely evolutionary patterns in each untreated CLL sample [20]. This analysis uncovered two possible evolutionary patterns. The majority of cases (20/24) were likely to have undergone branching clonal evolution where at least one subpopulation acquired genetic alterations giving rise to two or more distinct subpopulations. Four cases, however, accumulated the cytogenetic abnormalities probably in a stepwise manner consistent with linear evolution (Figure 2A). Samples displaying branching cytogenetic evolution could be further subdivided into those with simple subclonal branching (Figure 2B) and others with a more complex pattern characterized by the presence of multiple nodes (Figure 2C). CLL samples carrying both del(11q) and del(13q), the most common combination of cytogenetic abnormalities identified in our cohort (Figure 1A), exhibited both linear and branching patterns (Supplementary Table 2).

**Figure 3:**
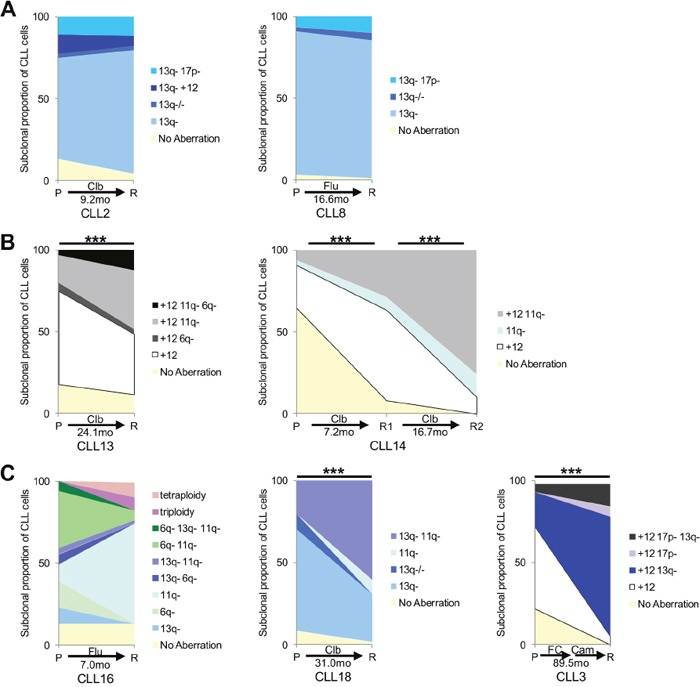
Multiplexed-FISH reveals differential subclonal responses to therapy in CLL Analysis of paired patient pre-treatment (P) and relapse (R) samples reveals different patterns of cytogenetic subclonal response. The observation time between each paired sample in months (mo) is shown. **(A)** Clonal stability with no change in the dominant clone between pre-treatment and relapse sample is observed in CLL2 and CLL8. **(B)** Clonal selection with a minor population in the pre-treatment sample becoming dominant at relapse is noted in CLL13 and CLL14. **(C)** Clonal evolution with the emergence of subpopulations carrying novel combinations of cytogenetic aberrations at relapse is evident in CLL3, CLL16 and CLL18. FC indicates fludarabine/cyclophosphamide; Clb: chlorambucil; Flu: fludarabine. Cytogenetic aberrations as denoted in Figures 1 & 2; 17p-: del(17p). Statistical significance denoted by ***P<0.001.

To determine changes in the composition of clinically relevant cytogenetic aberrations under the influence of different treatments, we analyzed 11 CLL cases by multiplexed-FISH in longitudinal samples taken before treatment and at relapse following either a single (n=8) or multiple (n=3) courses of chemotherapy, immunotherapy or chemoimmunotherapy (Table 1). We observed clonal stability in 4/11 cases with no change in the dominant subpopulation between pre-treatment and relapse (Figure 3A), and clonal selection in 7/11 cases where a minor subpopulation in the pre-treatment sample became dominant at relapse (Figure 3B). Of note, CLL2 and CLL8 harbored small subpopulations containing del(17p) but displayed clonal stability in response to chemotherapy. Although their CLL subsequently progressed, significant selection of subpopulations with del(17p) was not evident at relapse, suggesting that there may be other genetic events responsible for disease progression in these patients (Figure 3A). Interestingly, 3/7 cases with clonal selection also displayed clonal evolution, manifested by the emergence of CLL subpopulations carrying novel combinations of cytogenetic aberrations at relapse (Figure 3C). Given the limited number of cells assessed by multiplexed-FISH, it is plausible that these apparently novel subpopulations could have been present at very low frequencies in the pre-treatment sample and therefore evaded detection by multiplexed-FISH.

**Table 1:** CLL patient treatment and subclonal responses at relapse

Tumor	1^st^ Treatment	Response	2^nd^ Treatment	Response	Clonal Response
CLL2	**Clb**	PR	Flu	NR	Stable
CLL3	FC	PR	**Campath**	PR	Selection with evolution
CLL4	**FC**	CR	Ibrutinib	PR	Selection
CLL6	**FC**	PR	−	−	Stable
CLL8	**Flu**	PR	SCT	CR	Stable
CLL13	**Clb**	PR	**Clb**	PR	Selection
CLL14	**Clb**	CR	**Clb**	NR	Selection
CLL16	**Flu**	PR	SCT	CR	Selection with evolution
CLL18	**Clb**	CR	−	−	Selection with evolution
CLL19	**FC**	CR	Campath	CR	Stable
CLL32	**FC**	PR	FCR	PR	Selection

Paired pre-treatment and relapse samples of 11 patients were analyzed by multiplexed-FISH. Clonal response was determined by comparison of the subclonal architecture between pre-treatment and relapse samples. A ≥15% increase in a subclone size was considered indicative of clonal selection.

Note: Bold text indicates the treatment administered preceding the analysed relapse sample; Clb, chlorambucil; FC(R), fludarabine cyclophosphamide (rituximab); Flu, fludarabine; SCT, stem cell transplant; NR, no response; PR, partial response; CR, complete response.

Collectively, our results revealed distinct evolutionary patterns of cytogenetic lesions at the single-cell level. Different evolutionary patterns could be observed during disease progression in cases with initially similar cytogenetic composition.

### Clonal cytogenetic repertoire in CLL patients is broadly recapitulated in PDX models

It has previously been shown from PDX of acute leukemias that all leukemic subclones identified in patients display a propensity to engraft and proliferate in mice [23–27]. Conversely, the *in vivo* proliferative potential of CLL subpopulations with different genetic features is largely unknown. To investigate this, we generated PDXs using the pre-treatment sample from 7 CLL cases with multiple cytogenetic abnormalities. At 3-5 weeks following engraftment, FACS (fluorescence-activated cell sorting) sorted splenic CLL cells were analyzed by multiplexed-FISH (Supplementary Figure 1).

All CLL subpopulations in the samples from our cohort, identified by multiplexed-FISH based on their unique cytogenetic features, were capable of engrafting and proliferating in the PDX. However, in terms of subclonal similarity between patients and xenografts we observed two different trends.

**Figure 4:**
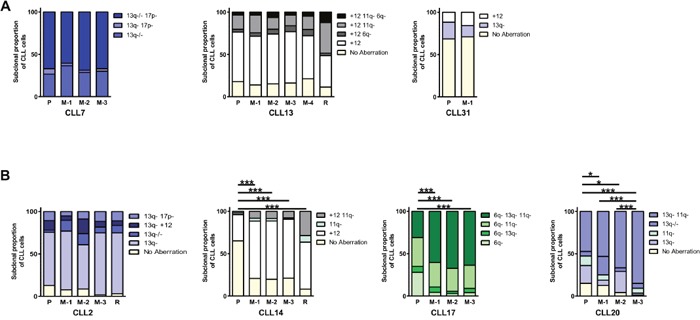
Recapitulation of patient cytogenetic profiles in CLL PDX models Irradiated (1.25Gy) NOG mice were intravenously co-injected with 2-5×10^7^ cells from a pre-treatment CLL sample and 1×10^5^ stimulated autologous T-cells. Multiplexed-FISH was used to assess the cytogenetic architecture of the patients’ pre-treatment sample (P), patients’ relapse (R) and FACS sorted cells from CLL PDX's (M) for seven tumors. This revealed that the subclonal cytogenetic composition of the input and engrafted cells could be either **(A)** highly similar (CLL7, CLL13, CLL31) or **(B)** significantly altered (CLL2, CLL14, CLL17, CLL20). M-1, M-2, M-3 and M-4 denote individual mice for each tumor (technical replicates). Cytogenetic aberrations are denoted as in Figures 1, 2 & 3. Statistical significance denoted by *P<0.05; ***P<0.005.

Notwithstanding minor variability across animals, in the PDX established for CLL7, CLL13 and CLL31 we observed that the clonal cytogenetic architecture in xenografts broadly resembled that of the pre-treatment CLL sample from which they were derived (Figure 4A).

In contrast, the cytogenetic architecture of CLL2, CLL14, CLL17 and CLL20 PDXs differed from the pre-treatment sample, indicating spontaneous clonal selection *in vivo*. In the CLL17 PDX, the selected subpopulation was the most cytogenetically complex, with concurrent deletions in 6q, 11q and 13q, whereas in the CLL14 PDX, two subpopulations, containing sole trisomy 12 and del(11q)/trisomy 12, respectively, were selected. In the CLL20 PDX, two of the three xenografts (M2/M3) displayed increased size of the del(11q)/del(13q) subpopulation, whereas the third xenograft (M1) displayed a four-fold increase in the biallelic del(13q) subpopulation (Figure 4B).

Importantly, in CLL14, the cytogenetic architecture of the PDX resembled that of the relapse sample (Figure 4B). For CLL17 and CLL20, relapse samples were not available, and therefore it was not possible to determine whether the cytogenetic architecture in these xenografts also resembled later stages of disease. In CLL2, there appears to be an expansion of the dominant subpopulation containing a sole del(13q) in 2/3 xenografts (M1/M3), such that the cytogenetic architecture was comparable to the relapse sample. However, unlike in CLL14, CLL17 and CLL20, the clonal changes in CLL2 were not statistically significant. The accelerated clonal progression in PDXs from CLL2 and CLL14 resembled chlorambucil-induced changes in the patients. Thus, it appears that in those cases post-treatment alterations may be spontaneous rather than treatment-driven, and possibly only accelerated by treatment. Of note, another PDX derived from a chlorambucil treated patient, CLL13, resembled the pre-treatment sample rather than the relapse sample. However, this patient had a longer progression free survival (PFS; 24.1 months) than CLL2 (9.2 months) and CLL14 (7.2 months) suggesting that the rapidity of the clonal changes in the PDX might reflect the disease course.

Collectively, our findings suggest that CLL PDX models broadly recapitulate the clonal cytogenetic repertoire observed in patients. Consistent with previous reports regarding well-established PDX models of ALL, our results also show that, in certain instances, clonal changes may occur at an accelerated rate in the PDX relative to the patient [28]. Many factors may contribute to this phenomenon including the efficiency of cell handling, transfer, homing and the balance between cell death and proliferation. However, it also appears that PDX clonal dynamics reflects the disease course as clonal shift broadly correlated with patients’ PFS. Thus, clonal changes in untreated xenografts reflect spontaneous clonal selection/evolution, and could provide a model for natural disease progression in individual patients.

### Multiplexed-FISH combined with PDX enables modelling of spontaneous and treatment-induced change in the CLL clonal cytogenetic architecture

Dynamic changes in the CLL clonal architecture can result from treatment-induced as well as spontaneous clonal progression. Having demonstrated that all CLL subpopulations detected by multiplexed-FISH can engraft in PDX models and that their dynamics can be captured, we next addressed the impact of different treatments on the clonal cytogenetic repertoire. We compared the cytogenetic evolutionary dynamics in response to DNA damaging agents (e.g. chlorambucil and fludarabine/cyclophosphamide (FC), monoclonal antibodies (e.g. rituximab) and clinically relevant small molecule inhibitors such as ibrutinib. To prevent excessive tumor load reduction which could hinder the likelihood of capturing the full range of subpopulations, including those at low frequencies, we treated CLL PDX models with reduced intensity therapeutic schedules. Our results revealed three different patterns of clonal dynamics.

**Figure 5:**
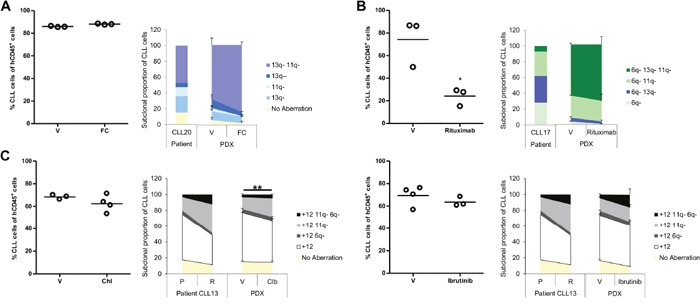
Effect of therapy on the cytogenetic architecture in CLL PDX models Subsequent to evidence that established PDX models were generated from 3 CLLs; usually one to two weeks after CLL/T-cell co-injection; they were treated under different protocols. Mice were sacrificed one week after fludarabine/cyclophosphamide, rituximab or chlorambucil treatment, or the day following cessation of ibrutinib treatment. Splenic tumor load was assessed by FACS analysis (left side of each panel). The cytogenetic architecture was derived from sorted splenic CLL cells (right side of each panel). **(A)** CLL20 PDX was treated with fludarabine and cyclophosphamide (0.625mg/kg and 6.25mg/kg, respectively; FC; n=3) or saline (vehicle; V; n=3) three times per week for two weeks. **(B)** CLL17 PDX was treated with rituximab (40mg/kg; n=3) or saline (V; n=3) three times over the course of a week. **(C)** CLL13 PDX was treated either with chlorambucil (5mg/kg; n=4) versus 3% DMSO (V; n=3) daily for five days (left panel) or with ibrutinib (12.5mg/kg; n=3) versus 1% methylcellulose/0.4% Cremephor EL (V; n=4) for nine days (right panel). Cytogenetic aberrations are denoted as in Figures 1, 2 and 3. Data are represented as mean ± SEM. Statistical significance denoted by *P<0.05; **P<0.01.

In CLL20, both untreated and vehicle-treated xenografts showed a significant difference in cytogenetic architecture compared to the pre-treatment patient sample, with selection of the del(11q)/del(13q) subpopulation (*P*<0.05; Figures 4B & 5A). More substantial selection of the same subpopulation was observed when xenografts were treated with FC (Figure 5A and Supplementary Figure 2). This finding supports the notion that chemotherapy can accelerate spontaneous selection for subclones with DNA damage response gene alterations such as those carrying del(11q), the *ATM* gene site. This in turn results in an inferior response to chemotherapy [13, 15].

On the contrary, in CLL17, all xenografts, whether vehicle-treated or rituximab-treated, displayed similar selection for the most complex subclone carrying all three abnormalities: del(6q), del(11q) and del(13q) (*P*<0.05; Figures 4B & 5B and Supplementary Figure 3). This is consistent with spontaneous clonal selection that occurred during xenotransplantation where no further substantive change in the cytogenetic architecture was observed upon treatment with monoclonal antibody. Whilst sub-optimal PDX treatments generally induced minimal tumor loss, rituximab caused a significant tumor reduction in PDX CLL17 suggesting that this aggressive and rapidly proliferating CLL was particularly susceptible to rituximab-mediated cytotoxicity.

Finally, a CLL13 PDX was established to compare the impact of two treatment modalities: chemotherapy and targeted therapy. The patient in this case was treated with chlorambucil and achieved a partial response. Subsequent relapse was characterized by differential outgrowth of the del(11q)/trisomy 12 subpopulation relative to the subpopulation carrying sole trisomy 12 (Supplementary Table 2 and Figures 4A & 5C). Chlorambucil-treated xenografts showed similar selection for the del(11q)/trisomy 12 subpopulation with a concomitant reduction in the subpopulation with sole trisomy 12 (*P*<0.01) (Figure 5C and Supplementary Figure 4). In contrast, CLL13 xenografts treated with ibrutinib showed a trend towards selection for the cytogenetically more complex subpopulation carrying trisomy 12 in combination with del(6q) and del(11q). These changes were not observed in the vehicle-treated xenografts (Figure 5C and Supplementary Figure 5). Thus, this PDX model demonstrated treatment-specific changes in the clonal cytogenetic repertoire.

Taken together, our results suggest that multiplexed-FISH in combination with CLL PDX models can potentially discriminate between the following types of changes in the clonal cytogenetic architecture: (a) spontaneous clonal progression that can be accelerated by treatment (e.g. CLL20 PDX); (b) spontaneous clonal progression that is unaffected by treatment (e.g. CLL17 PDX); and (c) treatment-induced clonal progression (e.g. CLL13 PDX).

## DISCUSSION

FISH remains the most widely used methodology for the evaluation of cytogenetic abnormalities in clinical practice despite the advent of targeted sequencing which is becoming more broadly available. The latter technique also requires substantial optimization regarding subclonal tracking in CLL PDX models. Previous studies have demonstrated that cytogenetic evolution in CLL predicts for inferior clinical outcome and frequently anticipates disease progression [2, 11, 12, 29–31]. However, both conventional FISH and targeted sequencing have limited value in the assessment of cytogenetic architecture or evolutionary dynamics because neither assesses multiple genetic aberrations within the same cell.

In this proof of principal study, we exploited the ability of multiplexed-FISH, a method that requires only a minor amendment of the standard laboratory technique, to identify and assess multiple, clinically relevant cytogenetic lesions within individual CLL cells. CLL is particularly amenable to such an approach due to the low number of recurrent cytogenetic aberrations, with only 7 lesions, namely del(13q), trisomy 12, del(11q), del(17p), del(6q), amp(2p) and del(8p), found to be present in more than 3% of CLL patients [32]. To demonstrate the utility of multiplexed-FISH, we focused on the first 5 of these lesions, which are among the most functionally significant cytogenetic aberrations in CLL. Del(17p13) results in the concomitant loss of *TP53* and *MIR3676*, with the former being essential for the DNA damage response, and the latter in inhibiting the CLL oncogene, *TCL1A*. In del(11q), the deleted region involves *ATM*, a DNA repair gene, and frequently *BIRC3*, a gene important for the regulation of non-canonical NFKB signaling. The loss of *DLEU2* (*MIR15A/16-1 cluster)* which occurs with del(13q14) results in the enhancement of CLL cell proliferation, whereas trisomy 12 is associated with *NOTCH1* mutations and increased integrin expression [33–36].

Due to the finite number of cytogenetic loci that can be assessed, multiplexed-FISH does not provide a comprehensive genome-wide catalogue of cytogenetic aberrations. Rather, by targeted analysis of a number of specific loci, it enables characterization of the relationships between functionally important cytogenetic aberrations in single CLL cells, from which CLL subpopulations can be readily identified. Hence, multiplexed-FISH provides an accurate and clinically relevant high resolution snapshot of the CLL cytogenetic architecture.

In most CLL studies to date, clonal dynamics have been inferred from bulk cellular populations. Here, we demonstrate that this can be directly observed at the single-cell level. Through analysis of longitudinal samples by multiplexed-FISH, we tracked temporal changes to the cytogenetic architecture in individual patients under different treatments. While in some instances no substantive change was observed, in other cases the cytogenetic architecture was perturbed by treatment, resulting in the selection of specific subpopulations, with or without an accompanying emergence of novel subpopulations (Figure 3). Due to a lack of sequential samples at earlier time-points, we were unable to directly examine pre-treatment evolutionary dynamics. Nevertheless, using the composition of cytogenetic aberrations in individual CLL cells, we were able to reconstruct possible evolutionary histories (Figure 2).

We showed that multiplexed-FISH can be readily applied to PDX models. Indeed, as we demonstrated in a series of proof-of-principle experiments, the application of multiplexed-FISH to PDX permits treatment-induced clonal changes to be distinguished from those occurring as a result of spontaneous clonal progression (Figures 4 & 5). We consider this to be a potentially important application for the pre-clinical evaluation of novel therapies. The outgrowth of tumor subpopulations that are less affected by treatment can give rise to eventual disease relapse. Hence, therapeutic efficacy should be measured not only in terms of the overall cytotoxic potential, but also of the ability to curtail CLL subpopulations with genomically destabilizing aberrations, thereby limiting genetic diversification and clonal evolution. In this respect, multiplexed-FISH can be used to monitor for spontaneous or treatment-induced changes of CLL subpopulations containing adverse cytogenetic features such as del(17p) or del(11q), as well as the emergence of cytogenetic complexity *in vivo*. This information may provide an insight into the likely nature and potential durability of treatment responses in patients with specific CLL cytogenetic compositions. Given the trend towards improved treatment stratification, this pre-clinical tool could potentially inform selection of appropriate patients into clinical trials.

In our proof-of-principle experiments, chemotherapeutic agents such as chlorambucil appeared to propel clonal selection, whereas treatment-induced clonal changes were not always observed with chemotherapy-free treatments (Figure 5). Although these results are by no means conclusive owing to the small sample size, they are however, broadly consistent with observations reported in clinical studies [13, 37–39] and confirm the mechanisms inferred by NGS [3, 40]. Thus, it remains to be determined whether this method allows the robust inference of general mechanisms broadly applicable to CLL. Future application of these experiments, initiated as part of a pre-clinical drug evaluation study, could encompass a more comprehensive panel of samples with different cytogenetic features.

There are a number of caveats to the use of PDXs and multiplexed-FISH in CLL [41]. Firstly, the level of tumor engraftment in CLL PDXs is lower than that of acute leukemias, which limits graft duration. Secondly, PDX models provide only a limited reflection of the CLL tumor microenvironment, as recipient mice are, by necessity, immunodeficient, and the murine stromal microenvironment is not identical to that in humans. However, we showed that this can be compensated to an extent by co-engrafting autologous T-cells, which also has the added benefits of improving engraftment efficiency and reflecting a facet of patient-related biology wherein CLL is also reliant on T-cells. In addition, the limited sensitivity of multiplexed-FISH imposes a restriction on the ability to distinguish between the appearance of novel and pre-existing subclones present below the limit of detection. However, it is important to note that the multiplexed-FISH results corroborate reported data from whole exome sequencing [32], a method with similar sensitivity (4%). Furthermore, although multiplexed-FISH provides a functionally important snapshot of the cytogenetic architecture and evolutionary landscape, it does not capture the full extent of subclonal dynamics and genomic aberrations, such as gene mutations. Therefore, future improvements may need to incorporate single-cell sequencing approaches to address such possibilities. Finally, manipulation of engraftment duration and timing of analysis for individual CLL cases might provide more faithful recapitulation of patient's clonal architecture and thus contribute to more precise assessment of treatment effects. Nevertheless, the significance of our study lies in the demonstration that treatment-induced clonal dynamics can be modeled *in vivo*, and these can be readily distinguished from spontaneous clonal progression, with important pre-clinical applicability.

In summary, this is the first report of a strategy to model CLL clonal dynamics *in vivo* that allows differentiation between spontaneous and treatment-induced clonal selection. FISH-based technology is well-established, validated, widely available, inexpensive and reasonably sensitive. Multiplexed-FISH provides a clinically relevant snapshot of the cytogenetic architecture at single-cell resolution. By applying multiplexed-FISH to PDX models, we provide proof-of-principle that they can be used to examine the evolutionary dynamics of cytogenetic aberrations at the single-cell level under different treatments. This can be applied to evaluate novel therapies in the pre-clinical setting, and to inform treatment choice for CLL patients with different cytogenetic composition, including those with adverse features or complex karyotype.

## MATERIALS AND METHODS

### Ethics statement

All studies involving clinical samples were approved by the UK National Research Ethics Service Committee West Midlands - Solihull and were conducted according to institutional guidelines and the principles expressed in the Declaration of Helsinki. Informed, written consent was obtained from all participants.

Investigations involving animals have been conducted in accordance with the ethical standards and according to the Declaration of Helsinki and according to national; United Kingdom Home Office; and international guidelines and have been approved by the UK National Research Ethics Service Committee West Midlands - Solihull.

### Patient samples

Samples were obtained from 128 CLL patients. Peripheral blood mononuclear cells (PBMCs) were sorted for CLL cells using a B-CLL cell isolation kit (Miltenyi Biotec; 130-093-661). Purity of sorted cells was assessed by flow cytometry and subsequent FISH analyses were corrected for the proportion of CLL cells in each sample by subtracting from the percentage of cells identified without cytogenetic aberrations.

### FISH analysis

FISH analysis was performed according to a standard protocol.[42] Whilst standard FISH uses probes with internal controls to interrogate individual loci, multiplexed-FISH employs bespoke probe combinations specific for each sample as previously determined by standard FISH. Cultured cell suspensions were harvested by treating with hypotonic solution (0.075M KCl; Sigma; P9541) for 15 minutes prior to fixation using a 3:1 mix of methanol: acetic acid (VWR International; 20847.307: Fisher Scientific; A/0360/PB170). Slides were exposed to 3.5μl probes; individual for standard FISH and simultaneous multiplexed-combinations for multiplexed-FISH (Supplementary Tables 1 & 2). Hybridizations were performed overnight on a HYBrite (Abbott Molecular) with an initial denaturation at 73°C for 2 minutes followed by incubation at 37°C for 16 hours. Cells were visualized on an Olympus BX50 microscope using a 100× objective. At least 200 cells were analyzed per patient sample. Samples were considered to have deletions of 11q, 6q, 13q, 17p or trisomy 12 if it was identified in more than 6%, 7%, 7.5% 7.5% or 3% of all analyzed cells, respectively, as determined by analysis of normal PBMCs. The proportion of cells with each deletion was compared between standard FISH and multiplexed-FISH and found to be similar. Subclones with multiple abnormalities were determined to be genuine, and not resulting from signal drop-out, if they were observed in ≥4% of a patient sample.

### Generation of PDX models and treatment administration

PDX models were established in 6-12 week old, male and female, NOD/Shi-SCID/IL-2Rγc^tm1sug^/Jic (NOG), *Mus musculus* Linnaeus, 1758 (mouse) strain animals (in-house) as previously described [43, 44].

Ibrutinib (Seleckchem; S2680) was resuspended in 1% methylcellulose and 0.4% Cremephor EL (Sigma; M0262 and C5135), and administered daily for 9 days by oral gavage at 12.5mg/kg, a dose which is sufficient to ensure 90% occupancy of Bruton tyrosine kinase (BTK) [45]. Rituximab (40mg/kg; Roche; 2530376), or saline control was given intravenously 3 times per week.[46] Fludarabine (0.625mg/kg, TEVA; 231-10-04151) and cyclophosphamide (6.25mg/kg, Baxter; 1001995501) or saline (control) were injected intraperitoneally 3 times per week, for two weeks, [47] whereas chlorambucil (5mg/kg; Sigma; CO253) or 3% dimethyl sulfoxide (DMSO, Sigma; D2650) vehicle control was administered daily for 5 days intraperitonally [48].

At specific time points (one week following treatment with chlorambucil, fludarabine/cyclophosphamide or rituximab and the next day following treatment with ibrutinib) animals were sacrificed and single cell solutions produced from their homogenized spleens. CLL cells were sorted by flow-cytometry as demonstrated in the Supplementary Figure 1.

### Statistical analysis

Statistical analysis was performed using unpaired Student's t-test or two-way ANOVA with Bonferroni *post hoc* comparison, *P*<0.05 was considered significant. Comparison of subclonal architecture of paired or serial patient samples was performed using the Chi-squared test, *P*<0.05 was taken to indicate clonal selection. All analyses were performed using GraphPad Prism version 5.0 (GraphPad Software, San Diego, CA).

## SUPPLEMENTARY MATERIALS FIGURES AND TABLES



## Abbreviations

ALL: acute lymphoblastic leukemia
amp: amplified
ANOVA: analysis of variance
BCR: B-cell receptor
BTK: Bruton tyrosine kinase
CLL: chronic lymphocytic leukemia
DMSO: dimethyl sulfoxide
del: deleted
FACS: fluorescent activated cell sorting
FC: fludarabine with chlorambucil
FISH: fluorescent *in situ* hybridization
NGS: next generation sequencing
NOG: NOD/Shi-SCID/IL-2Rγc^tm1sug^/Jic
PDX: patient-derived xenograft
PFS: progression free survival
