# Supplementary material for: Dynamic changes in clonal cytogenetic architecture during progression of chronic lymphocytic leukemia in patients and patient-derived murine xenografts
Source: Oncotarget. 2017 Apr 26;8(27):44749–60. doi: 10.18632/oncotarget.17432 (PMC5546515; doi:10.18632/oncotarget.17432)
Supplement: Supplementary file 1 [file oncotarget-08-44749-s001.pdf]

# Dynamic changes in clonal cytogenetic architecture during progression of chronic lymphocytic leukemia in patients and patient-derived murine xenografts

## SUPPLEMENTARY TABLES AND FIGURES

**Supplementary Table 1: FISH probes used in this study**

| Probe | Detects[a] | Company | Cat no. | Colour |
|---|---|---|---|---|
| **TP53/ATM** | 17p13.1/11q22.3 | Cytocell | LPH052 | Red/ Green |
| **CEP12** | Copy number chromosome 12 | Agilent | G100540B-8 | Aqua |
| **CEP12** | Copy number chromosome 12 | Cytocell | LPR012G | Green |
| **CEP10** | Copy number chromosome 10 | Cytocell | LPE010R | Red |
| **D13S319/CEP13** | 13q14.3/Copy number chromosome 13 | Cytocell | LPH0068 | Red/ Green |
| **D13S319** | 13q14.3 | Vysis | D13S319 (13q14.3) | Spectral Orange |
| **Myb/DEP6** | 6q23.3/Copy number chromosome 6 | Cytocell | LPH016 | Red/ Green |
| **Myb** | 6q23.3 | Vysis | LSI MYB | Aqua |
| **ATM** | 11q22.3 | Vysis | LSI ATM | Spectral Orange |
| **TP53** | 17p13.1 | Vysis | LSI TP53 | Spectral Orange |
| **TP53/CEP17** | 17p/Copy number chromosome 17 | Cytocell | LPH017 | Red/ Green |
| **ATM/ CEP11** | 11q22.3/Copy number chromosome 11 | Cytocell | LPH011 | Red/ Green |

[a]Cytogenetic aberrations detected by initial standard single probe FISH informed which probes to be multiplexed for each individual tumor and its associated PDX for multiplexed-FISH (Supplementary Table S2).

**Supplementary Table 2: CLL clinical characteristics and patterns of clonal evolution**

| Tumor | Binet Stage | TTFT, mo | 1st Treatment | Response | PFS, mo | Cytogenetics | Pattern of evolution | Multiplexed-FISH probe combination (colour)[a] |
|---|---|---|---|---|---|---|---|---|
| CLL1 | C | 2.7 | Clb | PR | 39 | del(11q),del(17p) | branching | TP53/ATM (R/G) |
| CLL2 | B | 0.9 | Clb | PR | 9.2 | +12,del(13q), del(17p) | branching | CEP12 (A), 13q/CEP13 (R/G), TP53 (Y) |
| CLL3 | C | 0.8 | FC | PR | 70.6 | +12,del(13q), del(17p) | linear | CEP12 (A), 13q/CEP13 (R/G), TP53 (Y) |
| CLL4 | A | 5.1 | FC | CR | 66 | +12,del(17p) | branching | CEP12 (G), CEP10 (R), TP53 (Y) |
| CLL5 | A | NT | - | - | - | del(13q) x2,del(17p) | branching | 13q/CEP13 (R/G), TP53 (Y) |
| CLL6 | A | 2.8 | FC | PR | 24.8 | del(13q),del(17p) | branching | 13q/CEP13 (R/G), TP53 (Y) |
| CLL7 | A | NT | - | - | - | del(13q),del(17p) | branching | 13q/CEP13 (R/G), TP53 (Y) |
| CLL8 | B | 1 | Flu | PR | 16.6 | del(13q),del(17p) | branching | 13q/CEP13 (R/G), TP53 (Y) |
| CLL12 | B | NT | - | - | -[b] | +12,del(11q) ,del(13q)x2 | branching | CEP12 (A), 13q/CEP13 (R/G), TP53 (Y) |
| CLL13 | A | 2.7 | Clb | PR | 24.1 | +12,del(6q), del(11q) | branching | CEP12 (G), CEP10 (R), 6q (A), ATM (Y) |
| CLL14 | A | 3.7 | Clb | CR | 7.2 | +12,del(11q) | branching | CEP12 (G), CEP10 (R), ATM (Y) |
| CLL15 | A | NT | - | - | - | +12,del(11q) | linear | CEP12 (G), CEP10 (R), ATM (Y) |
| CLL16 | C | 1.4 | Flu | PR | 7 | del(6q),del(11q), del(13q) | branching | 6q (A), ATM/CEP11 (R/G), 13q (Y) |
| CLL17 | A | 4.1 | Clb | CR | 30.8 | del(6q),del(11q), del(13q) | branching | 6q (A), ATM/CEP11 (R/G), 13q (Y) |
| CLL18 | A | 4.4 | Clb | CR | 31 | del(11q), del(13q)x2 | branching | ATM/TP53 (G/R), 13q (Y) |
| CLL19 | A | 4.6 | FC | CR | 10.5[b] | del(11q), del(13q)x2 | linear | ATM/TP53 (G/R), 13q (Y) |
| CLL20 | A | 10.2 | IbR | CR | 22.9 | del(11q), del(13q)x2 | branching | ATM/TP53 (G/R), 13q (Y) |
| CLL21 | B | 37 | FCR | CR | 4 | del(11q), del(13q)x2 | branching | ATM/TP53 (G/R), 13q (Y) |
| CLL22 | A | NT | - | - | - | del(11q),del(13q) | branching | ATM/TP53 (G/R), 13q (Y) |
| CLL23 | A | 7.9 | FCR | CR | 48.1[b] | del(11q),del(13q) | linear | ATM/TP53 (G/R), 13q (Y) |
| CLL24 | A | 3.7 | FCR | CR | 35.2[b] | del(11q),del(13q) | branching | ATM/TP53 (G/R), 13q (Y) |
| CLL25 | C | 0.3 | Clb | PR | 104.1 | del(11q),del(13q) | branching | ATM/TP53 (G/R), 13q (Y) |
| CLL31 | A | NT | - | - | -[b] | +12,del(13q) | branching | CEP12 (G), CEP10 (R), 13q (Y) |
| CLL32 | C | 0.1 | FC | PR | 20.7 | +12,del(13q) | branching | CEP12 (G), CEP10 (R), 13q (Y) |

[a]Combinations of multiplexed-FISH probes were ascribed to each individual tumor and its associated PDX based upon the cytogenetic aberrations initially detected by standard single probe FISH.
Note: TTFT indicates time to first treatment; mo, months; PFS, progression-free survival; NT, not treated; ; Clb, chlorambucil; FC(R), fludarabine cyclophosphamide (rituximab); Flu, fludarabine; IbR, ibrutinib with rituximab; CR, complete response; PR, partial response; [b]alive at time of censor; del, deletion of part of chromosome specified; +12, trisomy 12; .R, red; G, green; A, aqua; Y, yellow.

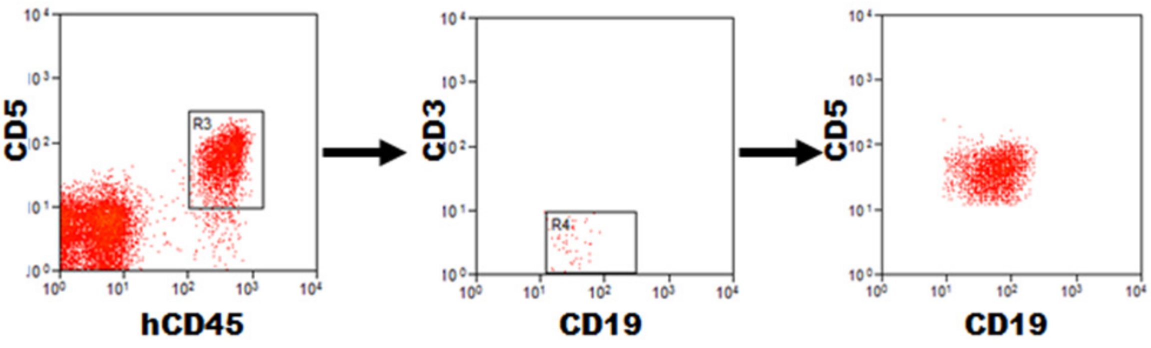


**Supplementary Figure 1: The gating strategy to isolate xenografted CLL cells from murine spleens.** Xenografted CLL cells were sorted from murine spleens using a Mo-Flo cell sorter based on expression of human CD45 (hCD45), hCD5, hCD19 and exclusion of CD3 and mouse (m)CD45 labeled with fluorescently-conjugated antibodies (eBioscience): hCD45-APC (9017-9459-120), hCD5-PerCP-Cy5.5 (9045-0058-120), hCD19-PE-Cy7 (25-0199-42), hCD3-APC-eFluor®780 (47-0036-42), mCD45-AlexaFluor®700 (56-0451-82). A representative image of a FACS sort is shown. Gate R3 identified $hCD45^+$ $CD5^+$ cells, gate R4 sorted the $CD19^+$ $CD3^-$ cells from those identified in R3. The right hand panel is the resultant $CD19^+$ $CD5^+$ sorted CLL cells. Their number represents the tumor load for a particular xenograft.

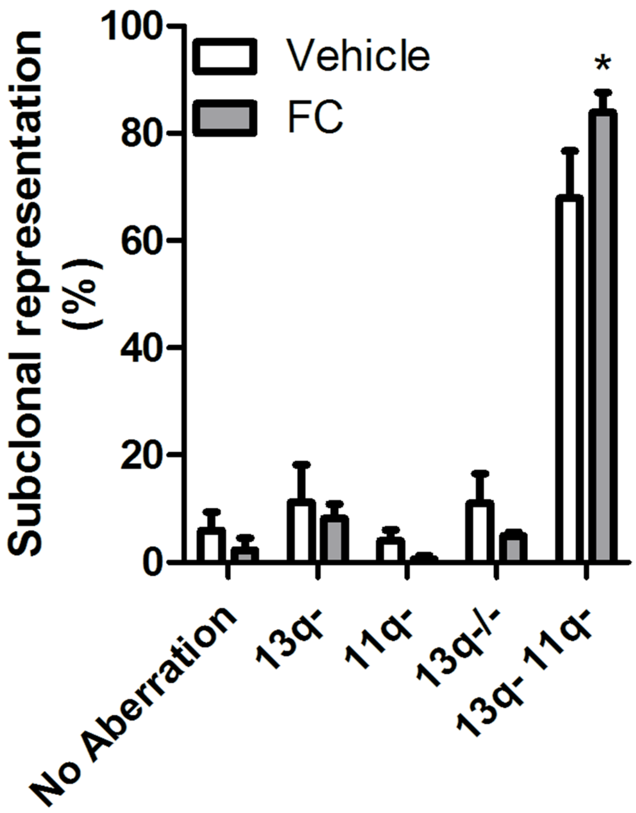


**Supplementary Figure 2: Assessment of FC therapy on cytogenetic subpopulations in a PDX of CLL20.** A PDX model of CLL20 was generated in 6 NOG mice. Engraftment was confirmed by flow cytometry and mice were randomised into treatment or control groups. Mice were treated with fludarabine/cyclophosphamide (FC; 0.625mg/kg and 6.25mg/kg, respectively) or vehicle three times per week for two weeks. CLL cells were isolated from spleens by FACS and cytogenetic subpopulations determined by multiplexed-FISH. The subclonal representation of the different cytogenetic subpopulations comparing the two treatment groups is depicted. 11q- indicates del(11q); 13q-: del(13q); del 13q-/-: del(13q)x2. Statistical significance denoted by *$P<0.05$. Error bars represent SEM.

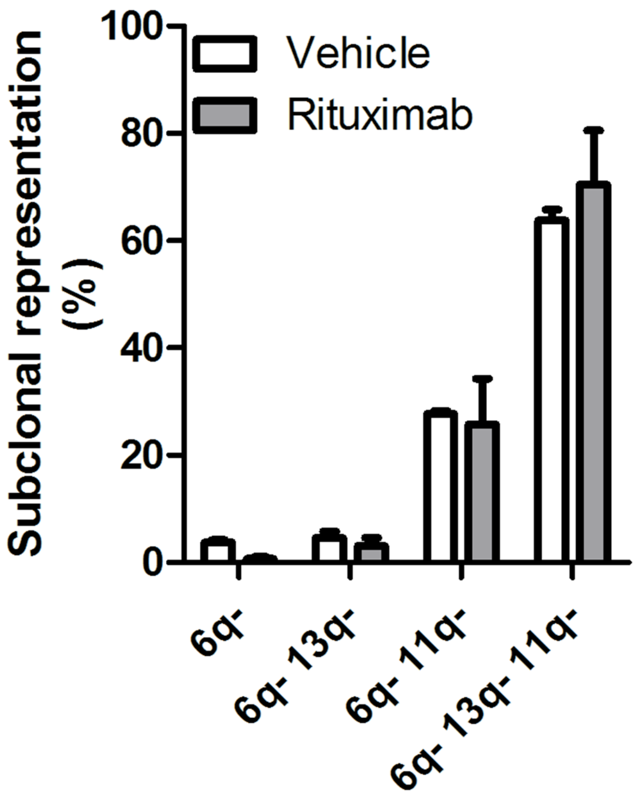


**Supplementary Figure 3: Comparison of the representation of specific cytogenetic subpopulations in response to rituximab therapy in a PDX of CLL17.** A CLL17 PDX was generated in 6 NOG mice. Engraftment was confirmed by flow cytometry and mice were randomised into rituximab or control groups. Mice were treated with rituximab (40mg/kg) or vehicle three times over the course of a week. CLL cells were isolated from spleens by FACS and cytogenetic subpopulations determined by multiplexed-FISH. The subclonal representation of the different cytogenetic subpopulations comparing the rituximab and vehicle treated groups is depicted. 6q- indicates del(6q); 11q-: del(11q); 13q-: del(13q). Error bars represent SEM.

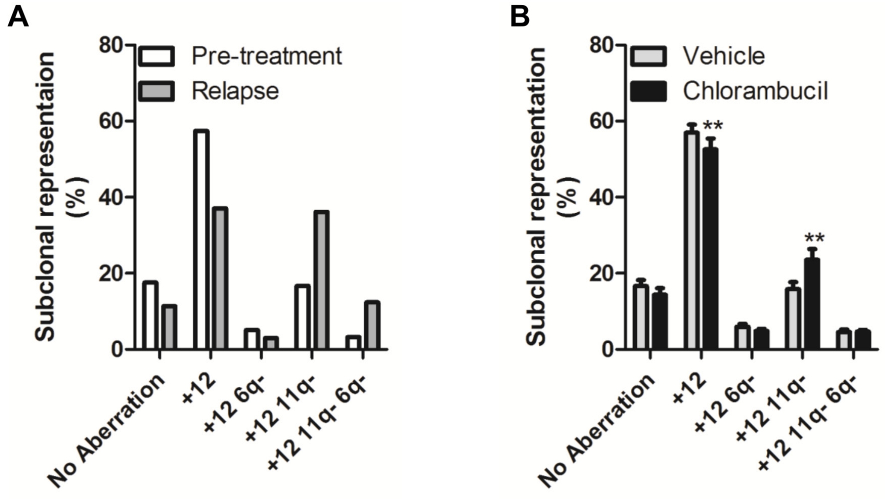


**Supplementary Figure 4: Analysis of patient and PDX cytogenetic subpopulations for CLL13. (A)** Comparison of the different cytogenetic subpopulations of CLL13, as determined by multiplexed-FISH, for pre-chlorambucil treatment and subsequent relapse. **(B)** A CLL13 PDX model was established and once engraftment was confirmed, mice were randomised into chlorambucil and vehicle treatment groups. Mice were treated daily with chlorambucil (5mg/kg) or vehicle for one week. CLL cells were sorted from murine spleens by FACS and cytogenetic subpopulations determined by multiplexed-FISH. The comparison of the subclonal representation of the different cytogenetic subpopulations between the two treatment groups is depicted. +12 indicates trisomy 12; 6q-: del(6q); 11q-: del(11q). Statistical significance denoted by $*P<0.05$. Error bars represent SEM.

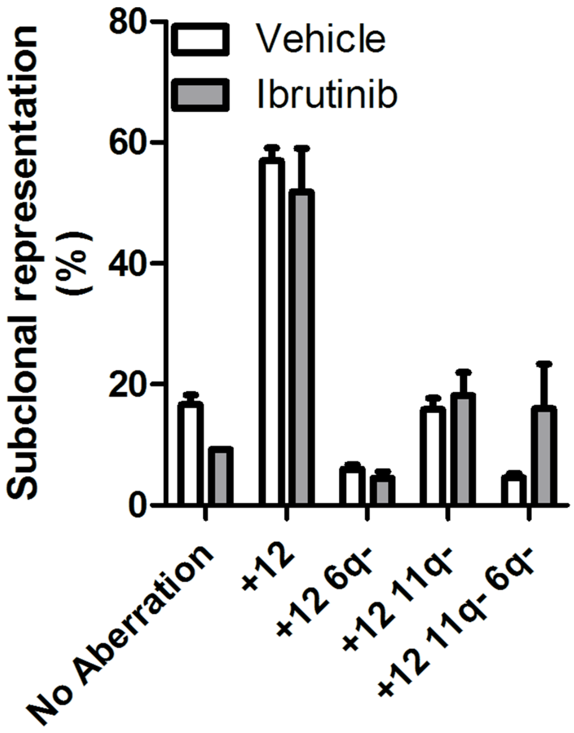


**Supplementary Figure 5: Analysis of CLL13 PDX cytogenetic subpopulation representation following ibrutinib treatment.** A CLL13 PDX was established and mice were split into two groups treated with either ibrutinib (12.5mg/kg) or vehicle for 9 days. CLL cells were isolated from the murine spleens using FACS and subsequently assessed by multiplexed-FISH. The representation of the different cytogenetic subpopulations comparing ibrutinib and vehicle treated groups is depicted. +12 indicates trisomy 12; 6q-: del(6q); 11q-: del(11q). Error bars represent SEM.
